# What to Do with the Insane

**Published:** 1891-09

**Authors:** F. S. White

**Affiliations:** Assistant Sup’t Terrell Insane Asylum; 1st Assistant Physician North Texas Hospital for the Insane, Terrell, Texas


For Daniel's Texas Medical Journal.

### WHAT TO DO WITH THE INSANE.

BY F. S. WHITE, M. D., 1ST ASSISTANT PHYSICIAN NORTH TEXAS HOSPITAL FOR THE INSANE, TERRELL, TEXAS.

THIS is one of the most important problems that to-day confronts the people of Texas; and it is one that must be squarely met and solved. It is important from two standpoints, that is, morally and financially. Humanity demands that this, the most unfortunate of our fellow beings, be comfortably cared for. The promptings of every man's higher senses force him to this conclusion. They are our brothers, and from no fault of their own, their moral and intellectual natures are destroyed or so perverted that they are incapable of earning for themselves a livelihood, and owing to this affliction are excluded from society. For this reason they appeal more strongly to the better and higher nature of mankind than any defective class of our population. These poor unfortunates, these miserable wrecks of noble men and women, cry for relief from the "felon's cell" of hundreds of cold, cheerless jails all over this fair land of ours. The miasmatic and noxious vapors arising from numbers of poorhouses scattered about over this great State mark the abode of hundreds of our demented fellow beings who at one time were stirred by ambition's subtle touch, and led on by the bright star of hope, which has set in their horizon to rise no more. "They have passed into a degeneration from over mental and emotional eminence in creation, to a state of perfect mindlessness, in which one is dead to the love and hatred, and to the joys and pains of life, oblivious to the past and unconcerned for the future. Stirred by no ambition, capable of no effort and unmoved by any motive, a mere human hulk like Byron's bark,

> "Thrown, when the war of winds is o'er,
> A solemn wreck on fortunes shore.
> 'Mid sullen, calm and silent bay,
> Unseen to drop by slow decay."

This condition of affairs is a reproach to the civilization of the latter quarter of the 19th century. The law makers evidently intended that all of the insane should be the wards of the State and be under her care and control, but the spirit of this law is not being obeyed, and why? It is not because the people of

Texas are less humane and sympathetic than in other and older States. It is not because they have any desire to neglect and to afford no relief to the insane. It is not because our legislators do not possess the spirit of philanthropy and do not desire to relieve the suffering of these poor creatures and add whatever of happiness lies in their power to their already disappointed lives, but it is because the people and legislators fail to grasp the enormity and magnitude of this charge upon the State. They cannot realize that there are in Texas to-day at the very least estimate, over four thousand insane, and that insanity is increasing more rapidly in proportion than our population. (I cannot now devote any time to the causes of this increase.) The ratio of the insane to the sane, for the entire United States, is one to five hundred and forty-five. In California, it is one to two hundred and sixteen. In the District of Columbia, one to one hundred and eighty-nine; New York, one to three hundred and sixty. If idiots be classed as insane, the ratio for the United States is raised to one to two hundred and sixty-nine. In States where the population is very near the same as that of Texas, the ratio of insane to sane ranges from 1 to 343, to 1 to 650. It can thus readily be seen that if the population of Texas be estimated at 2½ millions, that there are within her borders at a low estimate in round numbers 4000 insane and 5000 idiots. These calculations are based largely on the census of 1880. With this array of dependent population before us, it requires no argument to establish the fact that their care and support is no small matter, but one that demands immediate attention. It is very evident to every sane man that this class must be cared for. Their support is of course a tax on the more favored population and it devolves upon the State as the guardian and protector of her unfortunate citizens to provide for them in the best and most economical way possible. In devising ways and means to this end, it is expedient to take advantage of the experience of older and more advanced States in this line of charitable work. In New York all the insane (and she has an army of them 20,000 strong) have been made wards of the State, and additional buildings are now being erected to care for them and take them all out of the miserable jails and almshouses in which they have been so cruelly neglected for years, and give them comfortable homes where their lives, instead of being entirely a burden, may be rendered to a degree pleasant. Massachusetts, Pennsylvania and in fact all the older states are moving in the same direction. They realize the fact that in our advanced civilization this unfortunate class cannot longer be neglected. Then why should Texas, the empire State, longer refuse to make provision for all of her insane? If it is her duty to care for one of them, then it is equally her duty to care for all. It is a fundamental principle of every republican government that no discrimination shall be made between citizens. Texas has unlimited resources, and her people are not wanting in philanthropy, but as said above they do not realize the magnitude of this question. In her prosperity and greatness Texas must not forget and neglect her unfortunate citizens. Many who are now occupying the narrow confines of the "insane cells" of county jails and poor-houses, reeking in filth and disease, were once honored citizens of this commonwealth. Do not the dictates of every man's conscience tell him that they deserve better treatment? I have for several years been directly in contact with this class, and it is with sorrow and regret that I hear the pitiful supplications and appeals that are made by these people for a removal from the jails and poorhouses to the asylums. When the new building now in course of erection is completed, there will be accommodation in them all for only about 1700 insane, leaving at a low estimate 2300 outside.

There must be a reform in asylum construction. The day for palatial structures for the pauper insane I hope has passed. The main objects in erecting asylums should be safety and comfort, and this can be attained at a much less cost than heretofore. Where is the good sense or reason in placing a class of people who have always been accustomed to the simplest dwellings, in magnificent structures, fit for kings and queens?

Recent experiments in the provision for the insane have demonstrated the fact that asylums built on the cottage plan are the most economical and comfortable, and that the patients in such institutions are better and more comfortably cared for and are better satisfied. Now for something feasible. It is evident that Texas must do something for these people. They can, if properly managed, be made very largely self-sustaining. The State should purchase in some of her agricultural districts a large tract of land and erect thereon buildings on the cottage plan sufficient to accommodate 1000 insane. Let this be for the chronic more especially, but have a few wards for the acute cases. Let the asylums now built receive the acute from throughout the State, and transfer the chronic to this new asylum, where they can be utilized on the farm and in other works. An asylum of this character can be built at a cost of from $250 to $300 per bed. With a large tract of good farming land, and buildings of this kind, the insane could be made very useful in cultivating the land, and all sorts of industries could, at a trifling cost, be established that would give all who are able, employment. When constantly employed, either on farm or in shops, their health is much better, and a great pleasure is added to their lives, and their enforced confinement is rendered less irksome. The majority of them will take great interest in such work. The working classes furnish the greater number of the insane in our asylums, and it is a very valuable aid to treatment to be able to give them some good healthful outdoor employment. It not only does the body good, but takes the mind off their delusions and troubles and directs their thoughts into different and diverting channels. I believe that it is a safe assertion, that there are hundreds of asylum-made lunatics. Cases that are kept locked up in magnificent edifices, who become chronic by gazing day after day at snow white walls and polished floors. The tedious regularity of modern asylums soon becomes unbearably monotonous. This burdensome routine is detrimental to numbers of cases of insanity. Change is what is required, and the best manner of securing it is by giving the inmates diversified employment and by stimulating an interest in their work. Asylums founded on this plan are a success, both economically and from a therapeutic standpoint. There are so many insane in Texas, that it behooves the State to utilize their labor as far as possible to their support. This is best for the State from a business standpoint, and is best for the insane, for it gives them outdoor exercise, and as a consequence better health and happier lives, and lessens to a very great extent the burden of their affliction.

